# Early mitral valve repair surgery versus active surveillance in asymptomatic severe primary mitral regurgitation—insights from the Dutch AMR registry

**DOI:** 10.1007/s12471-025-02015-5

**Published:** 2026-02-09

**Authors:** Sulayman el Mathari, Einar A. Hart, Rosemarijn Jansen, Annemieke Wind, Jeroen Schaap, Maarten J. Cramer, Michiel L. Bots, Sebastian A. F. Streukens, Lodewijk Wagenaar, S. Matthijs Boekholdt, Mohamed Bentala, Jolanda Kluin, Steven A. J. Chamuleau

**Affiliations:** 1 Department of Cardiothoracic Surgery, Amsterdam University Medical Center, Amsterdam, The Netherlands; 2 Department of Cardiothoracic Surgery, Erasmus University Medical Center, Rotterdam, The Netherlands; 3 Department of Cardiology, University Medical Center Utrecht, Utrecht, The Netherlands; 4 Department of Cardiology, Saint Antonius Hospital Nieuwegein, Nieuwegein, The Netherlands; 5 Department of Cardiology, Diakonessenhuis Utrecht, Utrecht, The Netherlands; 6 Department of Cardiology, Amphia Hospital Breda, Breda, The Netherlands; 7 Dutch Network for Cardiovascular Research (WCN), Utrecht, The Netherlands; 8 Julius Center for Health Sciences and Primary Care, UMC Utrecht, Utrecht, The Netherlands; 9 Department of Cardiology, Maastricht University Medical Centre, Maastricht, The Netherlands; 10 Department of Cardiology, Medical Spectrum Twente, Twente, The Netherlands; 11 Department of Cardiology, Heart Center, Amsterdam Cardiovascular Sciences, Room Number G7-129, Amsterdam University Medical Center, 1105 AZ Amsterdam, The Netherlands; 12 Department of Cardiothoracic Surgery, Amphia Hospital Breda, Breda, The Netherlands

**Keywords:** Mitral valve regurgitation, Mitral valve surgery, Active surveillance, Early surgery, Facilitated surgery, Dutch AMR registry

## Abstract

**Background:**

Management of asymptomatic severe mitral regurgitation (MR) is challenging. Both early mitral valve repair surgery and active surveillance with facilitated surgery are possible strategies. The DutchAMR registry compares clinical outcomes between these two strategies.

**Methods:**

Patients were included between 2013–2019. Primary endpoints were cerebrovascular accidents (CVA), reoperations, and mortality. Facilitated surgery was defined as mitral valve repair surgery performed after developing a surgical indication during active surveillance.

**Results:**

Ninety-nine patients were enrolled; 71 in active surveillance and 28 in early surgery. Over a median follow-up time of 5.1 years, 51% of active surveillance patients underwent facilitated surgery due to ESC guideline triggers. Both the early and facilitated surgery groups had one perioperative CVA. During follow-up, in the active surveillance group, 5 (7%) patients died (3 without surgery and 2 after facilitated surgery), and 3 (4%) underwent reoperations. In the early surgery group, 4 (14%) patients reached a primary endpoint, including 2 (7%) CVAs (without residual symptoms) and 2 (7%) deaths. No reoperations occurred in the early surgery group. Baseline additional testing parameters based on CPET, Holter monitoring, and CMR showed no differences between the groups.

**Conclusions:**

After 5.1 years, half of the active surveillance patients required facilitated surgery, with comparable postoperative outcomes to early surgery. Clinical endpoints were comparable between the early and facilitated surgery strategies. There were no differences in baseline additional testing parameters, suggesting no clear targets for upfront stratificatio. Thus, shared decision making weighing the different risks can be used to determine the strategy per patient.

**Supplementary Information:**

The online version of this article (10.1007/s12471-025-02015-5) contains supplementary material, which is available to authorized users.

## What’s new?

- 51% of asymptomatic severe MR patients need facilitated surgery within 5 years.
- Early surgery and facilitated surgery (after active surveillance) have comparable clinical outcomes in patients with asymptomatic severe MR.
- All reoperations occur in the facilitated surgery group.
- Cerebrovascular accident events occur exclusive to the early surgery group.
- Baseline testing shows no correlation with primary outcomes in both groups.

## Introduction

Patients with severe primary mitral valve regurgitation (MR) may remain asymptomatic over extended periods, but once this balance is disrupted, clinical symptoms develop and worsen the prognosis due to associated adverse events [1–3]. Therefore, early diagnosis and management are key to improving outcomes. Guidelines recommend mitral valve repair (MVR) surgery for symptomatic patients with severe MR [4, 5]. For asymptomatic patients, surgery is advised if left ventricular ejection fraction (LVEF) is ≤ 60%, left ventricular end systolic diameter (LVESD) is ≥ 40 mm, and a durable repair is likely [4]. Class IIa indications include pulmonary hypertension, atrial fibrillation (AF), and left atrial dilation. However, the timing of surgery in asymptomatic patients with preserved LVEF remains a topic of debate [6–9].

Both early surgery and active surveillance are possible treatment strategies, with early surgery potentially preventing future LV dysfunction, while surveillance allows for timely intervention as needed [4, 10]. Literature shows contrasting outcomes: Rosenhek et al. found over 50% of patients were surgery-free after 8 years [6, 11], while Montant et al. reported that 90% required surgery within a similar timeframe [12]. And Suri et al. showed that early surgery is associated with improved survival [9]. These differences contribute to disparities in recommendations, with American guidelines favoring early MVR [10] and European guidelines favoring active surveillance [4].

The multicenter Dutch Asymptomatic Mitral Regurgitation (AMR) registry was established to compare early MVR and active surveillance in asymptomatic severe primary MR patients [13]. Patients were allocated to either strategy, and the registry aimed to (1) prospectively assess the rate of patients undergoing facilitated surgery during active surveillance, (2) elucidate whether facilitated surgery is non-inferior to early surgery in outcomes, and (3) explore whether baseline testing can help in selecting either strategy.

## Methods

The Dutch AMR study was initially a randomized controlled trial (RCT) for asymptomatic primary MR patients without surgical indication (ESC guidelines 2012), randomizing them to active surveillance or early surgery within 6 weeks after randomization to intervention [14]. Asymptomatic was defined as 1) having no complaints regarding exercise tolerance and normal exercise capacity measured by cardiopulmonary exercise testing (CPET). The primary outcome was a combined endpoint of cerebrovascular accidents (CVA), mitral valve (MV) reoperations, and cardiovascular and non-cardiovascular deaths. A registry arm included patients who opted out of randomization but consented to data collection. The study protocol has been published earlier [13] and was registered on clinicaltrials.gov (NCT01708265). Unfortunately, patient recruitment in the RCT arm was slow due to patient reluctance to undergo randomization for surgery, leading to the termination of the RCT in 2016. Consequently, it was decided to include all patients in the ongoing registry arm, including 12 already randomized patients. Of these 12 patients, 6 had already undergone surgery due to their allocation to the early surgery arm. The remaining 6 patients in the active surveillance arm have been given a new chance for allocation in the registry based on shared decision-making. The registry ended in 2020 due to insufficient enrollment. As such, the Dutch AMR registry consists of adult patients with severe primary MR, included between January 2013 and December 2019. Participating centers were 5 Dutch heart centers (Utrecht University Medical Center, Amphia Hospital Breda, Maastricht University Medical Center, Amsterdam University Medical Center, and Medical Spectrum Twente). This study was executed in compliance with the Declaration of Helsinki and has been approved by the institutional medical ethics committee (protocol number 12-120/E, date of approval 18-12-2012, Utrecht). Treatment strategies were determined following heart team consultation by shared decision-making. Written patient consent was obtained for pseudo-anonymous data collection from electronic files and phone consultations.

### Patient inclusion

Patient recruitment occurred at all participating centers. Prospective participants underwent assessments to confirm MR severity, evaluate preserved LV function, assess the likelihood of successful MVR, and determine operability. To minimize referral bias, both academic and non-academic hospitals were involved, encouraging recruitment of all asymptomatic MR patients [15]. Inclusion criteria included Holter monitoring to rule out paroxysmal AF and assess premature ventricular (PVC) and atrial contractions (PAC). Patients meeting all criteria (Electronic Supplementary Material [ESM] Tab. S1) were deemed eligible. Additional baseline testing, such as CPET and cardiac magnetic resonance imaging (CMR), was also encouraged for MR quantification and assessment of cardiac function and LV dimensions. All tests were conducted locally and collected centrally. Active surveillance involved vigilant annual monitoring for overt signs of deterioration (cardiac symptoms, LA dilatation, AF or PH), prompting facilitated surgery before LV dysfunction ensues.

### Study endpoints during follow-up

After inclusion, out-patient follow-up visits occurred at 3, 6, 12, 18, and 24 months, followed by annual checks. The evaluation focused on primary endpoints: 1) cerebrovascular accidents (CVA), 2) MV-related reoperations, and 3) cardiovascular and non-cardiovascular deaths, with non-retrievable causes classified as cardiovascular. Additionally, occurrences of AF and heart failure (HF) were assessed. If patients in the active surveillance group underwent MVR after developing a surgical indication, it was defined as facilitated surgery. Patient records were monitored until July 2022 to record outcomes.

### Statistical analysis

Continuous data is presented as mean ± standard deviation or median and interquartile ranges (IQR). Given the limited number of events and our small sample size, we present our data descriptively without conducting statistical tests. With this descriptive approach, we enhance the interpretability and accessibility of our results without the risk of overstating the significance of our findings.

## Results

**Fig. 1:**
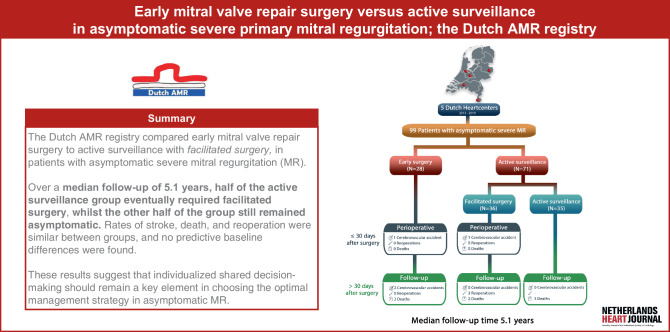
Infographic median follow-up time 5.1 years

In total, 99 patients were enrolled (77.8% male, mean age 56.9 ± 12 years). In 71 patients (77.5% male, mean age 57 ± 13), the initial strategy was active surveillance (of whom 6 patients were randomized in the RCT, and 65 patients were enrolled in the registry). And 28 (78.6% male, mean age 56.7 ± 10.7) underwent early surgery (6 patients in RCT, and 22 patients enrolled in registry setting). Left atrial volume index (mL/m^2^) was 51 for the active surveillance group and 46 for the early surgery group. The low sample size resulted in a low incidence of primary endpoints (Fig. 1; Electronic Supplementary Material [ESM] Tab. S2), leading to a lack of statistical power. Tab. 1 includes baseline characteristics for both groups.

**Table 1:** Baseline characteristics of the active surveillance and early surgery group included in the DutchAMR registry

Baseline characteristics		Active surveillance * n* *=* *71*	*Subgroup Facilitated surgery n* *=* *36*	*Subgroup Active surveillance remain asymptomatic during FU n* *=* *35*	Early surgery * n* *=* *28*
Age (years)		57 ± 13	55.2 ± 15	58.9 ± 11	56.7 ± 10.7
Male sex, *n* (%)		55 (78%)	29 (80.6%)	26 (74.3%)	22 (79%)
*Medical history*					
	Diabetes mellitus, *n* (%)	1 (1.4%)	0	1 (2.9%)	0
	Hypertension, *n* (%)	16 (23%)	5 (13.9%)	11 (31.4%)	4 (14%)
	Hyperlipidemia, *n* (%)	7 (10%)	3 (8.3%)	4 (11.4%)	1 (4%)
	Myocardial infarction, *n* (%)	1 (1.4%)	1 (2.8%)	0	0
	Coronary intervention, *n* (%)	1 (1.4%)	0	1 (2.9%)	0
	Cerebrovascular accident, *n* (%)	1 (1.4%)	0	1 (2.9%)	0
	Peripheral arterial disease*n* (%)	0	0	0	0
	Renal disease, *n* (%)	1 (1.4%)	0	1 (2.9%)	0
	Other valvular disease, *n* (%)	0	0	0	0
	Smoking, *n* (%)	10 (14%)	8 (22.2%)	2 (5.9%)	6 (21%)
	EUROSCORE II	0.58 (0.50–0.72)	0.61 (0.50–0.75)	0.5 (0.50–0.58)	0.61 (0.50–0.66)
*Laboratory values*					
	Hemoglobin (mmol/L)	9.1 (8.7–9.7)	9.1 (8.8–9.7)	9.1 (8.6–9.5)	9.3 (8.9–9.7)
	Creatinine (µmol/L)	79 (70–88)	79 (70–88)	77.5 (70.5–89.5)	81 (74–90)
*Echocardiographic data*					
	Left ventricular end-diastolic diameter (mm)	55 (50–58)	56 (50.3–58)	54 (48.5–58)	55 (48–60)
	Left ventricular end-systolic diameter (mm)	32 (29–36)	32 (30–36)	32.5 (29–37)	31 (25–39)
	Left atrial volume index (mL/m^2^)	51 (42–61)	51.7 (47–64)	46.2 (39–61)	46 (41–53)

Data is reported as number (%), mean (± standard deviation), or median (interquartile range)

### Active surveillance treatment strategy, including eventual facilitated surgery

During a median follow-up period of 5.1 years, 51% (36 out of 71) of patients in the active surveillance cohort underwent facilitated surgery, triggered by either symptomatic (*n* = 19, 53%) or asymptomatic (*n* = 17, 47%) indications for surgery. The symptomatic triggers included complaints of fatigue/dyspnea (*n* = 15), HF (*n* = 2), and endocarditis (*n* = 2) **(**Tab. 2**)**. Asymptomatic triggers were attributed to AF in 9 cases, LV dilatation in 3 cases, left atrial dilatation in 2 cases, the patient’s request in 2 cases, and pulmonary hypertension in 1 case. There were 5 deaths during active surveillance (2 in the facilitated surgery group and 3 in the group that did not undergo surgery). The facilitated surgery group included two cardiovascular deaths, and the remaining patients that did not undergo surgery included 2 cardiovascular and 1 non-cardiovascular (suicide) deaths.

**Table 2:** Reasons for facilitated surgery in the active surveillance population for both symptomatic and asymptomatic patient during the time of developing a surgical indication

Reasons for facilitated surgery in the active surveillance group			
	*Number of patients*		*Number of patients*
*Symptomatic*	19	*Asymptomatic*	17
– Dyspnea/fatigue	15	– Atrial fibrillation	9
– Heart failure	2	– LV dilatation	3
– Endocarditis	2	– LA dilatation	2
		– Patient’s request	2
		– Pulmonary hypertension	1

*LA* Left atrium, *LV* Left ventricle

### Postoperative primary endpoints facilitated surgery versus early surgery

Surgical characteristics were comparable between the facilitated surgery and early surgery group (Electronic Supplementary Material [ESM] Tab. S3). In the first postoperative 30 days, 2 patients reached a primary endpoint (1 CVA in each group), with both fully recovering and no postoperative AF (POAF) reported. There were no MV-related reoperations or deaths in this phase. During this same period, 8 patients in the facilitated surgery group experienced POAF, compared to 7 in the early surgery group. Follow-up, beyond the initial postoperative 30 days, showed AF incidence of 11% (4/36) in the facilitated group versus 7% (2/28) in the early group, with no HF reported.

**Fig. 2:**
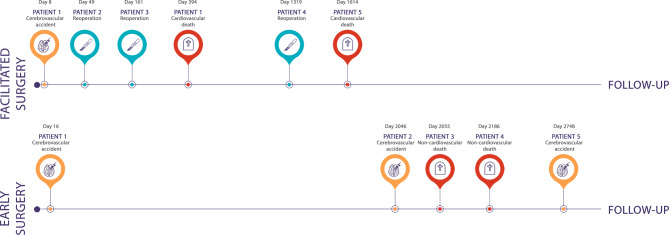
Timeline of primary endpoints in the facilitated surgery and early surgery groups

During follow-up (> 30 days after surgery), 5 patients in the facilitated surgery group reached a primary endpoint: 3 reoperations due to severe MR recurrence and 2 cardiovascular deaths. No CVA events were recorded in this group during follow-up. In the early surgery group, 4 patients reached a primary endpoint, including 2 CVAs and 1 cardiovascular, and 1 non-cardiovascular (amyotrophic lateral sclerosis) death. Both CVA patients fully recovered, and no AF was documented. There were no MV-related reoperations in the early surgery group. Reoperations within the facilitated surgery group were observed at 49,161, and 1318 days following surgery. The reoperation at 49 days was attributed to a recurrence of severe MR due to annuloplasty ring dehiscence. Reoperations at 161 and 1318 days were necessitated by new instances of MV prolapse. Tab. 3 provides an overview of the primary outcomes per subgroup, and Fig. 2 illustrates the timeline of all postoperative primary endpoints.

**Table 3:** Primary outcomes reported for all subgroups during both the perioperative (< 30 days after surgery) and the follow-up (> 30 days after surgery) phase

	*Early surgery n* *=* *28*	*Facilitated surgery n* *=* *36*	*Active surveillance remain asymptomatic during FU n* *=* *35*
*Perioperative (<* *30 days after surgery)*			
Cerebrovascular accident	1	1	–
Reoperation	0	0	–
Death	0	0	–
*Follow-up (>* *30 days after surgery)*			
Cerebrovascular accident	2	0	0
Reoperation	0	3	–
Death	2	2	3

### Additional testing at baseline & quality of life during follow-up

All patients underwent baseline testing (CPET, Holter monitoring, and CMR), with no differences between groups (Electronic Supplementary Material [ESM] Tab. S4). During follow-up, 89% of patients in the facilitated surgery group and 75% in the early surgery group reported well-being without cardiac complaints. One patient in the facilitated surgery and two in the early surgery group experienced palpitations. Three patients in the early surgery group versus one in the early surgery group, reported feeling worse postoperatively (fatigue). All patients under active surveillance reported well-being without complaints at the end of follow-up.

## Discussion

The findings of the multicenter Dutch AMR registry can be summarized as: (I) After a median follow-up time of 5.1 years, 51% of patients with asymptomatic severe MR developed a surgical indication and underwent facilitated surgery, (II) overall incidence of primary endpoints were 3 CVAs and 2 deaths in the early surgery group (*n* = 28) versus 1 CVA, 5 deaths and 3 reoperations in the active surveillance population (*n* = 71), (III) clinical endpoints were comparable between the early and facilitated surgery strategy, except for the incidence of CVAs during follow-up (only in the early surgery group; *n* = 2) and reoperations during follow-up (only in the facilitated surgery group; *n* = 3) and (IV) there were no differences in baseline additional testing parameters, suggesting no clear targets for upfront stratification.

These results corroborate the findings of Rosenhek et al. [6, 11], demonstrating that active surveillance can be safely accomplished in asymptomatic MR patients (*n* = 132) since half of the patients remained asymptomatic throughout the follow-up period, without meeting guideline indications for surgery. Additionally, the reoperation rate within the facilitated surgery group (5.3%) in this study was comparable to ours (8.3%). Our results, however, differ from a Belgian cohort (*n* = 192) where 90% of patients necessitated surgery within a median follow-up time of 8.5 years [12]. Additionally, the mortality rates in their active surveillance group (46.2%) and early surgery group (11.2%) were significantly higher than those observed in our cohort. These differences may be explained by the longer follow-up and the inclusion of patients with AF [16]; which was an exclusion criterion in our study. The largest cohort on this topic, comprising 2,097 patients [9], revealed improved survival rates for the early surgery group (86%) compared to active surveillance (69%), underscoring higher mortality rates compared to our study.

### Active surveillance patients who required facilitated surgery during follow-up

After half of the active surveillance group underwent facilitated surgery, all reoperations in our study were in this group, raising questions about whether delaying surgery affects MVR success (100% success in early surgery vs. 92% in facilitated surgery). The reoperations involved patients with Barlow’s disease (*n* = 1), anterior leaflet prolapse (*n* = 1), and posterior leaflet prolapse (*n* = 1). A possible explanation for the exclusive incidence of reoperations in the facilitated surgery group is that delaying surgery may allow for further progression of MV degeneration and LV remodeling. As severe MR persists over time, structural changes in the mitral apparatus and LV can occur, potentially making repair technically more challenging and increasing the likelihood of recurrent MR or repair failure. On the other hand, in clinical practice, early surgical intervention is often performed in patients with less advanced diseases and may therefore be associated with more durable repairs and a lower risk of reoperation. However, given the small number of reoperations in our study, the findings should be interpreted with caution and considered hypothesis-generating rather than definitive.

Furthermore, half of the active surveillance patients developing a surgical indication remained asymptomatic, and all patients developing AF reported no symptoms. This highlights the importance of vigilant monitoring [17].

### Perioperative and long-term outcome after early and facilitated surgery

In the perioperative period, one patient in each group experienced a CVA, with no POAF. Interestingly, both CVAs during follow-up (> 30 days post-surgery) occurred in the early surgery group, though no patients had residual symptoms or documented AF, raising questions about the cause of these events. Given the small sample size of this study, it is not possible to answer these questions, as these occurrences could also be attributed to chance. No perioperative deaths occurred, though two deaths in each group were recorded during follow-up.

Baseline tests (CPET ± VO2 max, Holter monitoring, CMR) showed no differences between groups, indicating similar preoperative status and limiting predictive stratification. Most patients in both groups reported good quality of life; however, a few (early surgery: 3; facilitated: 1) reported feeling worse postoperatively. All patients under continued active surveillance reported well-being without complaints, raising questions about the necessity of early surgery, as they may have remained asymptomatic without intervention. This suggests that subjective well-being, alongside further diagnostics, should play a role in shared decision-making on treatment strategies [18]**.**

### Limitations

Our study has limitations due to its small sample size, which hinders robust statistical analysis and requires cautious interpretation of results; therefore, the reported outcomes remain exploratory. Initially aiming for 250 participants, we faced difficulties in randomizing asymptomatic patients, leading to a low enrollment rate and making future RCTs unlikely. We may explore emulation target trial settings for better insights. Given the long timeline since the Dutch AMR study began, we opted to close the study and report outcomes based on the available sample [13]. Additionally, the lack of randomization may have introduced selection bias, as treatment strategies were chosen based on patient and cardiologist preferences. A further challenge is accurately determining the onset of severe MR, especially in asymptomatic patients who may be diagnosed incidentally. This could lead to an underestimation of the asymptomatic period before surgery. Another limitation is the incomplete collection of baselines NT-proBNP levels, which were missing for approximately 75% of study participants. As a result, we were unable to assess whether baseline NT-proBNP levels were associated with the likelihood of becoming symptomatic or the need for facilitated surgery during follow-up. Nor were we able to define the value of consecutive NT-proBNP measurements in this population.

This limitation stems from the pragmatic approach of our study, which followed routine clinical practice rather than a formal, protocol-driven research framework. While this approach increases the real-world relevance of our findings, it also led to incomplete data capture for certain variables, including biomarkers like NT-proBNP. In the absence of dedicated research funding or infrastructure, systematic testing and standardized follow-up were not consistently feasible. Consequently, our ability to perform detailed subgroup analyses or assess the value of additional prognostic indicators was limited. We acknowledge this as an inherent trade-off in pragmatic research, and it should be considered when interpreting our results.

Another important remark is that defining truly asymptomatic patients with severe MR is challenging, as clinical symptoms and AF are not always systematically assessed. Many previous studies have relied on observational data from registries or nonrandomized trials conducted in expert centers, where diagnostic protocols, such as Holter monitoring or exercise testing, were not consistently applied [19]. This lack of uniformity raises concerns about patient classification and may have influenced study outcomes, limiting the generalizability of results. Given the increasing prevalence of MR due to an aging population, it is crucial to adopt strict, standardized diagnostic criteria and refer all patients with severe MR to specialized heart teams for individualized evaluation. By adhering to guidelines and ensuring systematic assessments, we can improve both patient care and the quality of future research. As such, comparisons with prior studies should be made with caution.

## Conclusion

The incidence of primary outcomes in this study was low, but notable differences emerged between active surveillance and early surgery over a median follow-up of 5.1 years. Reoperations were only required in the facilitated surgery group, suggesting potential risks of delaying surgery. Conversely, all CVA events during follow-up occurred in the early surgery group, highlighting early intervention risks. However, these results should be interpreted with caution, as they may reflect random chance due to the small sample size. Furthermore, given that only half of the patients under active surveillance developed a surgical indication and mortality remained low, active surveillance appears to be feasible for asymptomatic severe MR patients. These results underscore that shared decision making weighing the different risks can be used to determine the strategy per patient with a asymptomatic severe mitral regurgitation and no class I or IIa guideline indication for surgery.

## Supplementary Information

Supplemental table 1. Inclusion and exclusion criteria used for the recruitment of patients for the DutchAMR registry. Supplemental table 2. Overview of all primary endpoints for the overall early surgery and active surveillance treatment strategy groups. Supplemental table 3. Surgical characteristics of both the facilitated surgery and early surgery group Supplemental table 4. Results of additional baseline testing at baseline for cardiopulmonary exercise testing, holter monitoring and cardiac magnetic resonance imaging for all three subgroups

This supplemental figure contains a flow diagram presenting the transition of the Dutch AMR Registry from randomized controlled trial to registry.

## Data Availability

Data will be made available upon request to the corresponding author.
