# Supplementary material for: Early mitral valve repair surgery versus active surveillance in asymptomatic severe primary mitral regurgitation—insights from the Dutch AMR registry
Source: Neth Heart J. 2026 Feb 9;34(3):90–9. doi: 10.1007/s12471-025-02015-5 (PMC12921052; doi:10.1007/s12471-025-02015-5)
Supplement: Supplementary file 1 — Supplemental table 1. Inclusion and exclusion criteria used for the recruitment of patients for the DutchAMR registry. Supplemental table 2. Overview of all primary endpoints for the overall early surgery and active surveillance treatment strategy groups. Supplemental table 3. Surgical characteristics of both the facilitated surgery and early surgery group Supplemental table 4. Results of additional baseline testing at baseline for cardiopulmonary exercise testing, holter monitoring and cardiac magnetic resonance imaging for all three subgroups [file 12471_2025_2015_MOESM1_ESM.docx]

# Supplementary files

**Supplemental table 1.** Inclusion and exclusion criteria used for the recruitment of patients for the DutchAMR registry. *ECG; electrocardiogram, ESC; European Society of Cardiology, RVSP; right ventricular systolic pressure*

| **Inclusion criteria** | |
| --- | --- |
|  | - Age 18–70 years |
|  | - Severe organic mitral regurgitation |
|  | - Absence of symptoms defined as absence of subjective limitations of exercise capacity or complaints expressed by the patient and confirmed by the cardiologist |
|  | - Likelihood of mitral valve repair should be >90% |
|  | - Patients should be fit for surgery |
|  | - Ejection fraction >60% and left ventricular end-systolic dimension <45 mm |
| **Exclusion criteria** | |
|  | - Class I or IIa indication for surgery according to the ESC guidelines [4] |
|  | - - Symptoms |
|  | - - Ejection fraction <60% or left ventricular end-systolic dimension >45 mm |
|  | - - Atrial fibrillation, either on 12-lead ECG or 48-h ECG monitoring |
|  | - - Pulmonary hypertension (RVSP >50 mmHg on echocardiography) |
|  | - Other life-threatening morbidity |

**Supplemental table 2.** Overview of all primary endpoints for the overall early surgery and active surveillance treatment strategy groups.

|  | **Early surgery** *(n=28)* | **Faciliated surgery** *(n=36)* | **Active surveillance** *(n=35)* |
| --- | --- | --- | --- |
| Cerebrovascular accidents | 3 (10.8%) | 1 (2.8%) | 0 |
| Reoperations | 0 | 3 (8.3%) | - |
| Deaths | 2 (7.1%) | 2 (5.6%) | 3 (8.6%) |
| *Data are presented as n (%)* | | | |

**Supplemental table 3.** Surgical characteristics of both the facilitated surgery and early surgery group.

| Surgical characteristics | | | ***Early  surgery*** *n = 28* | ***Facilitated  surgery*** *n = 36* |
| --- | --- | --- | --- | --- |
| EUROSCORE II | | | 0.61 (0.50-0.66) | 0.61 (0.50-0.77) |
| Surgical approach | | |  |  |
|  | Median sternotomy | | 21 (75%) | 26 (72%) |
|  | Lateral thoracotomy | | 7 (25%) | 10 (28%) |
| Cross-clamp time (min) | | | 97 ± 37 | 90 ± 36 |
| Perfusion time (min) | | | 128 ± 46 | 139 ± 59 |
| Annuloplasty ring size (mm) | | | 34 ± 3 | 36 ± 3 |
| Mitral valve repair | | | 27 (96%) | 35 (97%) |
| Mitral valve replacement | | | 1 (4%) | 1 (3%) |
|  | | Data are presented as means with standard deviation and n (%) | | |

**Supplemental table 4.** Results of additional baseline testing at baseline for cardiopulmonary exercise testing, holter monitoring and cardiac magnetic resonance imaging for all three subgroups.

|  | **Early surgery** *(n=28)* | **Facilitated surgery** *(n=36)* | **Active surveillance** *(n=35)* |
| --- | --- | --- | --- |
| **Cardiopulmonary exercise testing** | | | |
| VO_2_-max standard, *mL/kg/min* *(n)* | 24,6 *(6)* | 26,7 *(12)* | 24,4 *(11)* |
| VO_2_-max manual, *mL/kg/min* *(n)* | 30,2 *(11)* | 27,6 *(15)* | 27,7 *(16)* |
| **Holter** | | | |
| Premature atrial complexes, *% (n)* | 1,5 *(13)* | 0,63 *(18)* | 2,3 *(19)* |
| Premature ventricular complexes, *% (n)* | 0,7 *(13)* | 0,44 *(18)* | 0,46 *(19)* |
| **Cardiovascular magnetic resonance imaging** | | | |
| Left ventricular function, *% (n)* | 60,2 *(10)* | 59,6 *(15)* | 60,2 *(10)* |
| LVEDV, *ml (n)* | 266,6 *(10)* | 231 *(15)* | 225,7 *(10)* |
| LVEDV, *ml/m^2^ (n)* | 131,2 *(10)* | 123,2 *(14)* | 113,8 *(10)* |
| LVESV, *ml (n)* | 107,5 *(10)* | 86,2 *(15)* | 91,2 *(10)* |
| Stroke volume, *ml (n)* | 159 *(10)* | 133 *(15)* | 134,8 *(10)* |
| Abbreviations: LVEDV; Left ventricular end-diastolic volume, LVESV; Left ventricular end-systolic volume,  VO_2_; Maximal oxygen consumption  *Data are presented as means with standard deviation and n (%)* | | | |
