# Supplementary material for: Early mitral valve repair surgery versus active surveillance in asymptomatic severe primary mitral regurgitation—insights from the Dutch AMR registry
Source: Neth Heart J. 2026 Feb 9;34(3):90–9. doi: 10.1007/s12471-025-02015-5 (PMC12921052; doi:10.1007/s12471-025-02015-5)
Supplement: Supplementary file 2 — This supplemental figure contains a flow diagram presenting the transition of the Dutch AMR Registry from randomized controlled trial to registry. [file 12471_2025_2015_MOESM2_ESM.docx]

Supplemental Figures

**Supplemental Figure 1.** Flow diagram presenting the transition of the Dutch AMR Registry from randomized controlled trial tot registry.

**
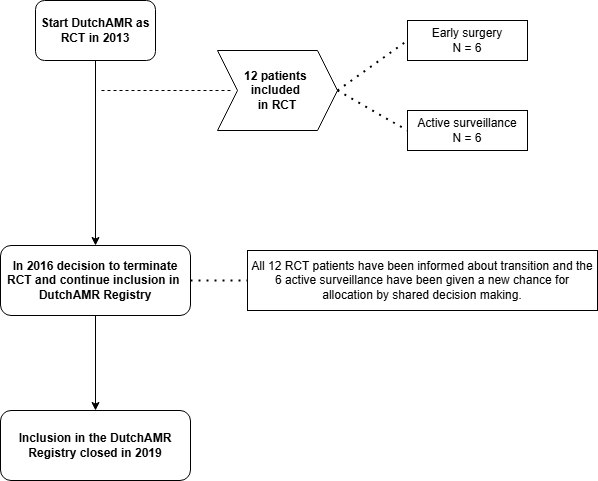
**
